# Morphometric and Molecular Analysis of Five-Spine *Epidinium* Morphotypes Taken from the Rumen of European Bison, *Bison bonasus*

**DOI:** 10.3390/life13122350

**Published:** 2023-12-15

**Authors:** Silvia Ivorová, Anna Kopčaková, Peter Pristaš, Svetlana Kišidayová

**Affiliations:** Institute of Animal Physiology, Centre of Biosciences, Slovak Academy of Sciences, 04001 Košice, Slovakia; kratochwillova@saske.sk (S.I.); kopcakova@saske.sk (A.K.); pristas@saske.sk (P.P.)

**Keywords:** rumen ciliates, *Epidinium*, five-spine morphotypes, European bison, phenotypic plasticity

## Abstract

An important feature of ruminal ciliates is their phenotypic plasticity, which makes their identification difficult. The common manifestation of the phenotypic plasticity in rumen ciliates is a change in their cell size and caudal spination. We analyzed various morphotypes of *Epidinium* with five caudal processes (spines) taken from the rumen of European bison (*Bison bonasus*). In the study, the cluster analysis and K-means analysis of morphometric data could not distinguish very similar morphotypes of *Epidinium* with five caudal processes. However, the morphotype of *E. parvicaudatum* prevailed (70%). The DNA of four individual *E. parvicaudatum* was isolated successfully from formaldehyde-preserved samples. The partial 18S rDNA gene sequences (about 350–400 bp) were identical to *Epidinium* sequences in GenBank (*E. caudatum*, a one-spine morphotype, and *E. cattanei*, a five-spine morphotype). It can be assumed that these short sequences cannot distinguish the differences between the *Epidinium* morphospecies. Complete gene sequences from various hosts and various molecular markers are necessary to reveal the validity of the *Epidinium* five-spine species. In conclusion, classical morphology should be supplemented with molecular data when more morphotypes of the rumen ciliate species are present in samples.

## 1. Introduction

A species concept is a working definition of a species or a methodology for determining whether or not two organisms are members of the same species. Scientists commonly use three species concepts. All species concepts have limitations and work better for some organismal groups than others. The biological species concept states that a species is a group of actually or potentially interbreeding natural populations reproductively isolated from other such groups. The lineage (phylogenetic) species concept defines species as groups of organisms that share a pattern of ancestry and descent and form a single branch on the tree of life. The morphological species concept is frequently applied when there is limited or unavailable data on reproductive behavior or genetic similarity. Morphological species (morphospecies) is the historical concept of species, accepted by many generations of naturalists, zoologists, and even today by many biologists [1,2,3,4,5]. Generally, two major approaches are used for the identification of ciliates: the traditional method of live-cell morphology, fixation, staining, and morphometrics, and, more recently, DNA-based methods [6,7]. Nowadays, taxonomy is more focused on an integrative approach combining classical and molecular approaches [1,8,9]. Phenotypic plasticity is the ability of an organism to change in response to stimuli or environmental inputs. The response may involve a change in physiological state, behavior, morphology, or some combination [10]. Ciliated protozoa colonize the forestomach (rumen) of all domestic and wild ruminants and pseudoruminants (llama, camel). Rumen commensal ciliates (Protista, Ciliophora, Litostomatea, Trichostomatia) take an active part in the fermentation processes in the rumen. We know more than 225 species of rumen ophryoscolecid ciliates described morphologically [11,12,13]. Their phenotypic plasticity makes identification difficult [14,15]. The common manifestation of the phenotypic plasticity in rumen ciliates is a change in cell size and caudal spination. *Entodinium*, *Ophryoscolex*, and *Epidinium* genera often react in this way. Under in vitro conditions, their spinated morphotypes gradually lose their caudal projections [16,17,18,19,20,21,22]. The genus *Epidinium* Crawley (1923) (family Ophryoscolecidae, order Entodiniomorphida) consists of spineless and spinated morphotypes. *E. capricornisi* [23], *E. cattanei* (syn. *E. ecaudatum* f. *fasciculus)* [24,25], *E. parvicaudatum* [12,25,26], and *E. rusa* [27] represent five-spine morphospecies between which confusion may occur in ecological studies [11,28]. They differ in the size and shape of their cell body and spines. In addition, there is a discrepancy in the description and names of *E. parvicaudatum*, *E. cattanei*, and *E. fasciculus* among some authors [11,16,24,25,26,28,29,30]. In some studies, the descriptions and names of Dogiel monography are preferred [12,16,30]. However, *E. fasciculus* is not a valid name for *E. cattanei* [11,12,29]. There has been much discussion about whether similar epidinia, which differ only in the number of caudal spines, are different species [11,25,29]. Dogiel suggested that epidinia with varying numbers of spines are morphological variations (forms) of one species, *E. ecaudatum* [25], a spineless morphotype. On the other hand, the “ecaudatum group” was established with *Epidinium ecaudatum* as a type species characterized by an elongated body tapering posteriorly with no spine by Kofoid and McLennan [29]. The group consists of morphotypes with 0–5 caudal spines, e.g., *E. caudatum*, *E. bicaudatum*, *E. tricaudatum*, and *E. parvicaudatum*. On the contrary, *E. hamatum*, *E. cattanei*, and *E. bulbiferum* differ from the ecaudatum group by the body’s thick and heavy posterior part. Spinated forms also occur there [29]. *E. cattanei* (syn. *E. ecaudatum* f. *fasciculus*) and *E. parvicaudatum* (syn. *E. ecaudatum* f. *parvicaudatum*) are the relatively common five-spine morphotypes. *E. cattanei*, Fiorentini (1889), is characterized by its relatively short body and five long, straight caudal spines in contrast to *E. parvicaudatum*, Awerinzew and Mutafowa (1914), which is characterized by body tapering posteriorly with five incurved, heavy caudal spines of which the ventral is the largest [11,12,24,25,26]. In the rumen of European bison, *Epidinium* morphotypes with 0–5 spines occurred there [31]. Five-spine morphotypes prevailed, and they were assigned to *E. parvicaudatum*. However, other morphotypes were observed, and many individuals were similar to *E. cattanei*. In the present study, the authors hypothesized that different morphotypes might be differentiated by a multivariate analysis of morphometric data. To date, only two *Epidinium* morphotypes, *E. caudatum* (syn. *E. ecaudatum* f. *caudatum*, one-spine morphotype) and *E. cattanei* (syn. *E. ecaudatum* f. *fasciculus*, five-spine morphotype) have been molecularly analyzed [30,32]. *E. cattanei* formed a distinct group from *E. caudatum* [6,30,32]. We isolated the DNA from individual *Epidinium* five-spine morphotypes of European bison samples preserved by formaldehyde to resolve their molecular taxonomy.

## 2. Materials and Methods

The study focused on analyzing the five-spine morphotypes of *Epidinium* of the rumen ciliate population of the European bison (*Bison bonasus*). In February and March 2007, rumen content samples were obtained from 18 culled European bison in Białowieża Forest, Poland [31]. Samples were collected immediately after death (1–3 h). Approximately 5 g of the rumen content from each animal was preserved with an equal amount of 8% formaldehyde. Preserved content was filtered through four layers of gauze and transported to the Institute of Animal Physiology of the Slovak Academy of Sciences in Slovakia, where they were kept in the fridge [31].

### 2.1. Morphometric Analysis

The pictures of *Epidinium* five-spine morphotypes were taken under bright-field illumination by a Moticam Pro CCD Camera (Motic Incorporation Ltd., Hong Kong) mounted on a BA400 microscope (Motic Incorporation Ltd., Hong Kong). The wet rumen samples on microscope slides were colored by Lugol’s iodine solution. We have analyzed the set of 272 pictures of *Epidinium* from 15 European bison. We manually measured the ciliate length and width on the calibrated pictures using ImageJ (1.52a, Wayne Rasband, National Institutes of Health, Kensington, MD, USA). The spines were not included in the length measurement because they had a variable length. We measured the length in the median plane from the apical pole to the caudal end. The width was measured where the cell was the widest (usually in the middle plane) and the line was perpendicular to the length line. The ratio of cell length to width was calculated. The morphometric data (length and width) were statistically evaluated in Microsoft Excel (Microsoft Office 2016 Professional Plus), IBM SPSS, version 19 (IBM Corporation, Armonk, NY, USA), and PAST, version 4.04 software [33]. IBM SPSS was used for the Kolmogorov–Smirnov normality test. The multivariate analysis of morphometric data similarity (clustering and K-means analysis) was carried out according to the PAST guide [33]. The orientation of the epidinia is described according to the position of skeletal plates. It is said that the cytostome with an adoral ciliary zone (AZM) and skeletal plates are on the ventral side of the ciliate [11,34]. The dorsal ciliary zone (DZM) and two contractile vacuoles are on the dorsal ciliate side. When the ciliate is rotated with the skeletal plates on the right side, the upper side of the image can be assigned to the right side, and the lower side of the image can be given to the left side of the ciliate. In this study, we use the original two-word nomenclature of individual morphotypes and, in parentheses, the equivalent terminology, according to Dogiel [11,25].

### 2.2. Molecular Analysis

*DNA isolation.* To resolve the taxonomy of the five-spined epidinia, the DNA was isolated from 25 individual five-spine *Epidinium* with a long ventral caudal spine from the formaldehyde-preserved rumen fluid of European bison. DNA from individual protozoal cells was isolated by the chelex method [35]. The individual ciliate cell was picked out from the drop of formaldehyde-preserved rumen fluid under a microscope using a micromanipulator fitted with a glass capillary. The cell was washed three times in 10 µL of distilled sterile water on a glass slide and transferred to a mini tube filled with 50 µL of 5% Chelex-100 (BIO-RAD, Hercules, CA, USA) in water and vortexed for 15 s. Pre-incubated proteinase K (Merck, Rahway, NJ, USA) was then added to the reaction solution to a final concentration of 10 µg/mL and incubated for 20 min at 98 °C). After incubation, the solution was cooled to 0 °C, centrifuged (10,000 rpm, 10 min, and 4 °C), and the supernatant was used directly as a template for PCR amplification.

*Polymerase-chain-reaction.* For PCR amplification of the 18S rDNA gene, two primer sets synthesized by Sigma-Aldrich, St. Louis, MO, USA) were used. The primer sets EukFor (5′-AATATGGTTGATCCTGCCAGT-3′) and EukRev (5′-TGATCCTTCTGCAGGTTCACCTAC-3′) were able to amplify the nearly full-length 18S rRNA gene (of about 1500 bp), and primer sets Cill-R-GC (5′-CGCCCGCCGCGCCCCGCGCCCGGCCCGCCGCCCCCGCCCGGGGCCAATTGCAAAGATCTATCCC-3′) and Cill-F (5′-GGTGGTGCATGGCCG-3′) were able to amplify the first 400 bp of the 18S rDNA gene (Moon van der Stay, unpublished). PCR amplification was carried out using a C1000TM Thermal Cycler (BIO-RAD, Hercules, CA, USA). For amplification of the full-length 18S rDNA gene, the conditions specified by Regensbogenova et al. were used [27]. However, the original method was unsuccessful for PCR amplification [27]. The modified method was as follows: The Cill-R_GC and Cill-F primer sets were used for amplification. The PCR reaction was performed as follows: initial denaturation (94 °C, 4 min), 35 cycles of denaturation (94 °C, 1 min), annealing (55 °C, 30 s), extension (72 °C, 1 min), denaturation (94 °C, 1 min), annealing (65 °C, 30 s), and elongation (72 °C, 10 min). Electrophoresis in 1% agarose gel and 100 bp ladder as standard of molecular size were used to determine the quality and quantity of the obtained PCR products. Wizard SV Gel and PCR Clean-Up System (Promega Corporation, Madison, WI, USA) were used to purify PCR products. The concentration of DNA was measured by a NanoDrop 2000c spectrophotometer (Thermo Fisher Scientific Inc., Colorado Springs, CO, USA). Obtained amplicons were sequenced using the same primer pair for amplification at Eurofins Genomics (Ebersberg, Bayern, Germany). Obtained sequences were assembled using DNA Baser ver. 2.0 (Heracle BioSoft SRL, Cluj-Napoca, Romania) and analyzed using the Blastn algorithm against the GenBank database. For phylogenetic comparisons, the 18S rDNA sequences of *Epidinium* spp. and related species were downloaded from the GenBank database and aligned using the Clustal W algorithm. The neighbor-joining tree was constructed using the MEGA software version 7 with 1000 bootstrap repetitions [36]. The list of the sequences of *E. parvicaudatum* of European bison are summarized in Appendix A, Table A1.

## 3. Results

### 3.1. Morphometric Analysis

**Figure 1:**
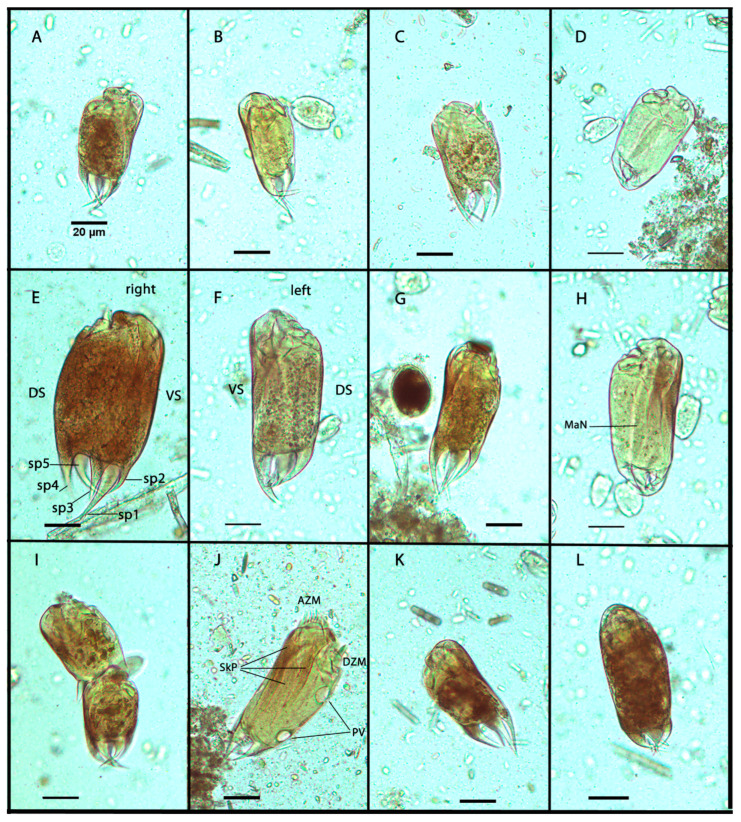
Photomicrographs of five-spine morphotypes of *Epidinium* from the rumen of European bison (*Bison bonasus*). Samples were colorized with an iodine solution. The protozoan right side (**A**,**D**,**E**,**H**,**K**); the protozoan left side (**B**,**C**,**F**,**I**,**J**); the protozoan dorsal side (**G**,**L**); a dividing ciliate (**I**). The typical representatives of the *E. parvicaudatum* morphotype (**B**,**E**,**F**,**J**). DS, dorsal side; VS, ventral side; sp1–sp5, the numbered sequence of spines; MaN, the macronucleus; AZM, adoral zone of membranels; DZM, dorsal zone of membranels; SkP, skeletal plates; PV, pulsatile vacuoles. The length of the scale bar is 20 µm in all pictures.

About 84% of epidinia cells with five spines had cylindrical bodies with wide body ends (Figure 1A,C–F). About 16% of epidinia were slightly tapering posteriorly (Figure 1B,J). The cells were either straight or slightly bent on the ventral side. The dorsal side was usually more convex when viewed from the right or left side. According to the shape of the spines, four morphotypes can be distinguished in the rumen of European bison. Five incurved, heavy caudal spines, of which the ventral was the largest, as is typical for *E. parvicaudatum*, were observed in 70% of individuals. (Figure 1A,B,E,F). They had a specific arrangement of spines: a single largest spine (sp1) on the ventral surface, two spines on the right surface (sp2 and sp3), a single spine on the dorsal surface (sp4), and a single spine (sp5) on the left surface (Figure 1E), [18]. The second morphotype (3%) had spines incurved at an angle greater than 80 degrees, usually towards the opposite side (Figure 1D,H). About 11% of epidinia had caudal spines of approximately the same length, the morphotype similar to *E. cattanei*. (Figure 1C,G,K). About 16% of epidinia had five short, incurved spines similar to *E. rusa* (Figure 1L). No individuals with furcated spines (*E. capricornisi*) were observed. The two contractile vacuoles on the dorsal side were regularly visible in the opening stage (Figure 1J). Epidinia had three skeletal plates on the ventral side that were typically well visible after staining with an iodine solution (Figure 1J). When epidinia were full of starchy feed, the ciliates were too dark to distinguish the intracellular organelles after iodine staining. The morphometric descriptive parameters (length, width, and ratio of length to width) of the five-spine *Epidinium* cells are summarized in Table 1. The values of the parameters are as follows: The length (L) varied from 67.5 µm to 144.4 µm, and the mean value was 103.1 µm. The width (W) ranged from 35.9 µm to 89.9 µm; the mean value was 57.4 µm. The ratio of L/W varied from 1.34 to 2.36, and the mean value was 1.8.

**Figure 2:**
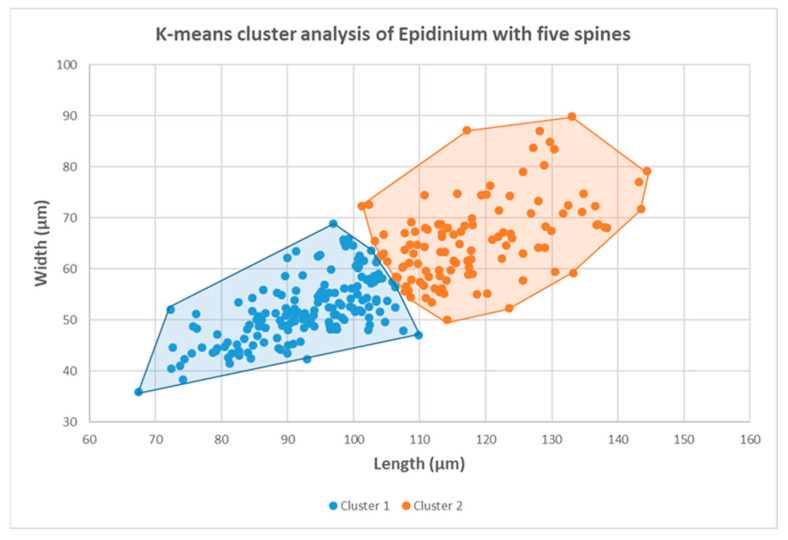
The clustering of the five-spine morphotypes of *Epidinium* from the rumen of European bison (*Bison bonasus*) by K-means analysis.

The K-means clustering analysis revealed the two main clusters in the set (Figure 2). The groups differ by ciliate size. Smaller epidinia (60%) were clustering in cluster 1. In total, 40% of epidinia were clustering in cluster 2. Epidinia in both clusters had the same mean ratio of L/W (1.8).

All morphological features described above were present in both groups and each animal (various spines and endings of the body). There were also dividing cells in both groups. In the group of smaller ciliates (cluster 1; Figure 1A–D), 23% of ciliates were in different dividing stages. In cluster 2 (larger ciliates, Figure 1E,F,H,J,L), 37% of ciliates were in the different dividing stages. The omitting of dividing ciliates from clustering had no substantial effect on the K-means analysis. The authors observed 29% of the morphotype of *E. parvicaudatum* (long ventral spine) in cluster 1 and 41% in cluster 2, with a prevalence of the wide-ending ciliate bodies. The morphometric descriptive parameters of both groups are summarized in Table 2.

### 3.2. Molecular Analysis

**Figure 3:**
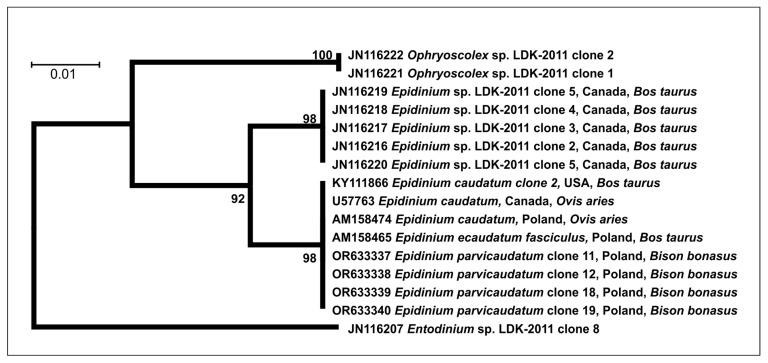
Rooted neighbor-joining tree showing relatedness of *Epidinium parvicaudatum* (syn. *E. ecaudatum* f. *parvicaudatum*) partial 18S rDNA sequences obtained through this study to the related rumen ciliate sequences in GenBank database. Numbers at nodes are bootstrap values after 1000 repetitions. As an outgroup, the 18S rDNA sequence of *Entodinium* sp. LDK-2011 clone 8 was used.

To resolve the taxonomy of the five-spine epidinia, the DNA was isolated from 25 individuals of five-spine *Epidinium* with a long ventral caudal spine from the formaldehyde-preserved rumen fluid of European bison. Only four samples were successfully sequenced. No full-length 18S rRNA PCR amplicons from formaldehyde single-cell *Epidinium* sp. samples were obtained using the EukFor and EukRev primer pair. Using primer-pair targeting, the first 400 nucleotides of the 18S rDNA gene (Cill) yielded specific amplicons from four cells. The phylogenetic analysis of the sequences is presented in Figure 3. These short sequences of the 18S rDNA gene clustered together with *Epidinium* sequences in GenBank (*E. caudatum*, a one-spine morphotype, and *E. cattanei*, a five-spine morphotype) isolated from the ruminal content of several ruminants from geographically distant localities (Figure 3). Short sequences of unidentified *Epidinium* sp. (JN116217-220) clustered in different group.

## 4. Discussion

This study focused on analyzing the five-spine morphotypes of *Epidinium* from the rumen ciliate population of the European bison. Based on the morphometry and microscopic observation, it is hard to decide if the observed *Epidinium* five-spine morphotypes could be separate species. However, no individuals with furcated spines (*E. capricornisi*) [2,14,19] were observed in European bison. In the study, other five-spine morphospecies were observed. They resembled *E. parvicaudatum* (70%), *E. cattanei* (11%), and *E. rusa* (16%). There was an as-yet undescribed form with spines curved inward at an angle greater than 80 degrees (3%). These morphological variations were observed in all European bison examined. It is the first observation of the simultaneous coexistence of four morphotypes of five-spine epidinia. It is an observation of the remarkable plasticity of rumen ciliates. Similarly, Dehority observed the remarkable plasticity of *Entodinium dubardi* from the blue duiker [6]. He observed several forms of *E. dubardi* similar to other non-spine *Entodinium* species. Polymorphism was also observed in the species of *Diplodinium* and *Ophryoscolex* [5,7,8]. According to previous descriptions, *E parvicaudatum* has been detected worldwide in ruminants (cattle, gaur, sheep, goats, and giraffes) [2]. *E. cattanei* has been described from cattle, zebu, sheep, and goats worldwide [2]. *E. rusa* has been described from sambar deer in India [18]. Compared to the previous descriptions of *E. parvicaudatum* and *E. cattanei* [2], the dimensions of the five-spine epidinia were in the range of both morphotypes. The K-means analysis provided the clustering of epidinia according to size. We can speculate if these clusters represent the two different species; all morphological features used to distinguish epidinia species (various spines and endings of the body) were present in both groups. The populations of ruminal protozoa of the same morphotype differing in cell size were observed in some ecological studies [22]. The large and small *Eudiplodinium maggii* populations were observed in the rumen of European bison [22]. It is supposed that it could be a reaction to predation. Entodinia, epidinia, and other ciliates are objects of predation by other rumen ciliates, usually by *Polyplastron*, *Elytroplastron*, and *Entodinium bursa* [11,31,37,38,39,40]. The epidinia were predated by *Elytroplastron bubali* in European bison [22]. Therefore, the larger size may be a strategy to prevent predation. It is consistent with the assumption that larger and broader ciliates resist predation better. It can be said that the variability of spines is not surprising. It is the primary protection against predation. The ability to form different spines probably allows for better survival of the species. In addition, ciliates without a long spine may represent a population of upper daughter cells after the division of the mother cell (Figure 1I). During division, short caudal spines were visible in upper daughter cells before separation. The spines likely grow after the cells have separated. However, the presence of a long barb seems to be advantageous, as this form was the most numerous. The plasticity of the spination was proven during cultivation experiments. Clarke reported that *Epidinium* mixed culture (individuals with 1–5 spines) lost all caudal spines after seven months in vitro [12]. Coleman et al. reported a similar loss of the caudal spine in *Epidinium caudatum* culture after 4–5 months in vitro [10]. Dehority used clonal cultures of *E. caudatum* (one-spine form), *E. tricaudatum,* and *E. parvicaudatum* [9]. During a short period (about seven days), he reported bifurcated and trifurcated spines in epidinia and changes in the counts of spines. In the study of Miltko et al., the length of spines of *E. cattanei* (syn. *E. ecaudatum* f. *fasciculus*) only decreased after 12 months in vitro [8]. It seems that the five-spined form was relatively stable. The original and other descriptions of *E. parvicaudatum* describe long ciliates with posteriorly tapering cell endings and *E. cattanei* as relatively short, wide ciliates [2,15]. The morphotype description contrasts our observations, where most ciliates with a long ventral spine had wide bodies along their entire length. The tapering of the cell end does not appear to be a valid character for classifying *Epidinium* morphotypes, nor does the size and shape of the spines. For now, the authors are inclined to believe that the different five-spined morphotypes in European bison probably do not represent separate species.

The molecular taxonomy of European bison five-spine *Epidinium* was carried out to resolve their taxonomical relationship. According to the authors, this is the first report on the isolation of DNA from samples of rumen ciliates preserved by formaldehyde. The lack of amplification of the full 18S rDNA gene was probably due to extensive DNA fragmentation during formaldehyde-preserved sample storage [41]. The DNA fragmentation extracted from formalin-preserved paraffin-embedded specimens (FFPE) is well known. Formaldehyde functions as a cross-linking agent for cell (tissue) preservation. It stabilizes the tissue (cell) structure by creating a covalent linkage between macromolecules, such as DNA–DNA, DNA–protein, and protein–protein [42]. Reversing the formaldehyde-formed cross-linking during DNA extraction causes DNA fragmentation [42]. The degree of damage is affected not only by the preservation procedure but also by the storage time and storage conditions [43]. The significant degradation of DNA was observed after 4 to 6 years of sample storage [43]. On the other hand, DNA isolated from FFPE samples kept at a low temperature (4 °C) was less fragmented than DNA from samples kept at laboratory temperature [44]. However, many studies have demonstrated the feasibility of using FFPE DNA for next-generation sequencing, including gene-specific targeted sequencing, whole-exome, and whole-genome sequencing [45]. Lin et al. [46] successfully amplified DNA from FFPE samples. They assumed that DNA from FFPE samples with fragments less than 200bp may be suitable for genetic studies. Even though long-term storage of the European bison samples led to intense DNA degradation, we obtained shorter PCR amplicons. In our study, using a primer pair targeting the first 400 nucleotides of the 18S rDNA gene (primers Cill-R_GC and Cill-F) and modifying PCR reactions yielded specific amplicons from four cells. The main changes in the PCR protocol were made in the annealing and extension phases.

The sequences of *Epidinium* of European bison were identical to several *Epidinium* isolates in the GenBank. However, it is possible that the short sequences cannot distinguish the differences between the *Epidinium* morphospecies. It cannot be excluded that epidinia from European bison are genetically distinct from other *Epidinium* morphospecies. In addition, the sequences of unidentified *Epidinium* sp. (JN116217-220) clustered in another group. They are only short partial sequences of the 18S rDNA gene. No morphometric description can be found on the *Epidinium* sp. Until now, only the 18S rDNA genes have been used in the molecular analyses of the genus *Epidinium* and other rumen ciliates. Furthermore, few *Epidinium* species have been sequenced. To date, the complete sequences of 18S rDNA genes of the species *E. caudatum* (*E. ecaudatum* f. *caudatum*) and *E. cattanei* (syn. *E. ecaudatum* f. *fasciculus*) are available [21,23]. Different *Epidinium* morphotypes were not molecularly analyzed. Phylogenetic analysis of the complete sequences of 18S rDNA genes showed differences between *E. caudatum* and *E. cattanei* [21,23]. The full-length 18S rRNA sequences of the one-spine morphotype (*E. caudatum*) differ from the five-spine morphotype (*E. cattanei*) by 12 nucleotide substitutions (data not shown). Identifying and classifying the organisms based on conserved and variable regions of 18S rDNA is a standard procedure in taxonomy studies of eukaryotic microorganisms [47]. 18S rDNA genes are highly conserved intra-species markers among eukaryotes, and finding potential primer sites specific to certain taxa can be challenging [48,49]. The internal transcribed spacer regions (ITS1 and ITS2) that separate nuclear ribosomal genes are considered to be quite variable and have helped discriminate interspecific and intraspecific genetic variation from a wide diversity of life from bacteria to plants and vertebrates. Among rumen ciliates, the ITS1 and ITS2 regions were used to discriminate variations between eight isolates of *Isotricha prostoma* from two continents (North America and Australia) and different hosts (sheep and cattle). No differences were reported, indicating that the regions are 100% conserved [50]. Other rumen ciliates were not evaluated by this marker. No other markers were used in the evaluation of the phylogenetic relationship of rumen ciliates. The ITS1 and ITS2 were used in the phylogenetic analysis of other free-living litostomatean ciliates [51]. The single- and multilocus analyses of ITS1-5.8S rDNA-ITS2 were in agreement with previous 18S rDNA gene phylogenies, supporting that both 18S rDNA and ITS region sequences are effective tools for resolving phylogenetic relationships among the litostomateans [51]. The single- and multilocus analyses were also used in the phylogenetic study of endocommensal astome ciliates from earthworms. Their systematic positions were determined using the 18S rDNA gene, ITS1-5.8S-ITS2region, and the barcoding D1/D2 domains of the 28S rDNA gene [52].

## 5. Conclusions

The morphometric and molecular analysis results are inconclusive to decide whether the five-spine *Epidinium* from the European bison represents one or more species. For now, it can be recommended to name the *Epidinium* morphotypes according to Dogiel [25]. Thus, the spination will be included in the name; although, it is unclear how this feature relates to the phylogenetic relationship of these morphotypes. Complete sequences from various hosts are necessary to reveal the validity of the *Epidinium* five-spine morphospecies. Using other potential molecular markers (large ribosomal DNA subunit—LSU rDNA, 28S rDNA) and internal transcribed spacers (ITS1 and ITS2) can produce a more reliable view of the relationships between rumen ciliate morphospecies [1,2,8,9,53,54,55,56].

## Figures and Tables

**Table 1:** Descriptive statistics of the body length, body width, and the ratio of body length to body width of the five-spine rumen ciliate *Epidinium* from European bison.

*Epidinium*	Length (µm)	Width (µm)	L/W
Mean	103.1	57.4	1.8
Standard Error	0.96	0.60	0.01
Median	101.29	56.02	1.80
Standard Deviation	15.71	9.70	0.18
Minimum	67.45	35.93	1.34
Maximum	144.42	89.90	2.36
Count	266	266	266

**Table 2:** Descriptive statistics of the body length, body width, and the ratio of body length to body width of the five-spine rumen ciliate *Epidinium* from the European bison after K-means clustering.

	Cluster 1			Cluster 2		
	Length (µm)	Width (µm)	L/W	Length (µm)	Width (µm)	L/W
Mean	92.2	51.7	1.8	118.7	65.8	1.8
Standard Error	0.73	0.49	0.01	1.01	0.76	0.02
Median	93.73	51.18	1.80	116.14	65.16	1.83
St. Deviation	9.39	6.30	0.17	10.69	8.05	0.20
Minimum	58.7	33.5	1.4	101.24	50.00	1.34
Maximum	109.9	68.9	2.3	157.49	89.90	2.36
Count	166	166	166	112	112	112

## Data Availability

The datasets used and analyzed during the current study are available from the corresponding author upon reasonable request.

## Appendix A

**Table A1:** Accession numbers of the partial sequences of the 18S rDNA gene of the rumen commensal ciliate *Epidinium parvicaudatum* (syn. *Epidinium ecaudatum* f. *parvicaudatum*) isolated from the formaldehyde-preserved rumen samples of European bison (*Bison bonasus*).

Species Name	Isolate Label	GenBank Accession Number	Collection Year	Country of Origin of the Host	Locality	Host Organism	Source
*E. parvicaudatum* (Sharp, 1914)	Clone 11	OR633337	2007	Poland	Bialowieza Forest	*Bison bonasus* (Linnaeus, 1758)	rumen
*E. parvicaudatum* (Sharp, 1914)	Clone 12	OR633338	2007	Poland	Bialowieza Forest	*Bison bonasus* (Linnaeus, 1758)	rumen
*E. parvicaudatum* (Sharp, 1914)	Clone 18	OR633339	2007	Poland	Bialowieza Forest	*Bison bonasus* (Linnaeus, 1758)	rumen
*E. parvicaudatum* (Sharp, 1914)	Clone 19	OR633340	2007	Poland	Bialowieza Forest	*Bison bonasus* (Linnaeus, 1758)	rumen
